# Impact of Baseline Muscle Mass and Myosteatosis on the Development of Early Toxicity During First-Line Chemotherapy in Patients With Initially Metastatic Pancreatic Cancer

**DOI:** 10.3389/fonc.2022.878472

**Published:** 2022-05-20

**Authors:** Sun Hong, Kyung Won Kim, Hyo Jung Park, Yousun Ko, Changhoon Yoo, Seo Young Park, Seungwoo Khang, Heeryeol Jeong, Jeongjin Lee

**Affiliations:** ^1^ Department of Radiology and Research Institute of Radiology, Asan Medical Center, University of Ulsan College of Medicine, Seoul, South Korea; ^2^ Biomedical Research Center, Asan Institute for Life Sciences, Asan Medical Center, Seoul, South Korea; ^3^ Department of Oncology, Asan Medical Center, University of Ulsan College of Medicine, Seoul, South Korea; ^4^ Department of Statistics and Data Science, Korea National Open University, Seoul, South Korea; ^5^ School of Computer Science and Engineering, Soongsil University, Seoul, South Korea

**Keywords:** pancreatic cancer, myosteatosis, muscle mass, chemotherapy, toxicity

## Abstract

**Objectives:**

Although chemotherapy is the only treatment option for metastatic pancreatic cancer (PDAC), patients frequently encounter adverse events during chemotherapy leading deterioration of patients’ quality of life and treatment interruption. We evaluated the role of baseline CT-assessed body composition in predicting early toxicity during first cycle of the first-line chemotherapy in patients with metastatic PDAC.

**Methods:**

This retrospective study included 636 patients with initially metastatic PDAC who underwent first-line chemotherapy from January 2009 to December 2019. Chemotherapy regimen, baseline laboratory data, and body composition parameters acquired from baseline CT were obtained. The skeletal muscle index (SMI) was used to identify patients with a low muscle mass (SMI < 41 cm^2^/m^2^ for women, and < 43 cm^2^/m^2^ [body mass index < 25 cm/kg^2^] or < 53 cm^2^/m^2^ [body mass index ≥ 25 cm/kg^2^] for men), and myosteatosis was defined as low-attenuated muscle area divided by skeletal muscle area (LAMA/SMA index) ≥ 20%. Univariate and multivariable binary logistic regression analyses were performed using bootstrapping with 500 interactions to identify predictors of grade 3–4 toxicity and any treatment-modifying toxicity which led to a dose reduction, delayed administration, drug skip or discontinuation.

**Results:**

During the first cycle of the first-line chemotherapy, grade 3–4 toxicity and treatment-modifying toxicity occurred in 160 patients (25.2%) and in 247 patients (38.8%), respectively. The presence of both low muscle mass and myosteatosis was significantly associated with the occurrence of both grade 3-4 toxicity (odd ratio [OR], 1.73; 95% confidence interval [CI], 1.14–2.63) and treatment-modifying toxicity (OR, 1.83; 95% CI, 1.26–2.66) whereas low muscle mass alone did not.

**Conclusions:**

The presence of both low muscle mass and myosteatosis assessed on baseline CT may be used to predict early chemotherapy-related toxicity in patients with metastatic PDAC.

## Introduction

Metastatic pancreatic ductal adenocarcinoma (PDAC) is well known for its dismal prognosis, with the median overall survival is less than 1 year and the 5-year survival rate is only 3% (1). Chemotherapy is the only treatment option which can improve survival for patients with metastatic PDAC (2), and FOLFIRINOX (5-fluorouracil, oxaliplatin, irinotecan, and leucovorin) and gemcitabine-based regimen are currently accepted as the first-line therapy (3). However, both regimens carry unfavorable toxicity profiles (4) which could ultimately result in treatment interruption, and a considerable portion of patients require dose modification, treatment delay or cessation in both regimens due to toxicity (5). As the treatment-related adverse events lead to deterioration of patients’ quality of life and treatment interruption leading to unfavorable prognosis and poor survival (6–9), it would be important to predict those who are likely to experience chemotherapy-associated toxicity.

Sarcopenia, defined as the loss of muscle quantity and strength (10), has recently gained attention as a potential predictor of survival or treatment-associated complications in multiple types of cancers (11–13). In addition, myosteatosis, the fatty degeneration or infiltration of the muscle (14), is related to decreased muscle strength and quality (15) and has also emerged as a potential predictor for treatment-related adverse events and survival in cancer patients (12, 13, 16–18). Computed tomography (CT) is the most widely used tool to assess body composition by estimating the areas of each tissue using CT attenuation values (19, 20). In terms of muscle evaluation, beyond the mere quantification of the muscle mass, CT can evaluate the quality of muscle by assessing myosteatosis.

Several studies have shown the impact of sarcopenia and myosteatosis on the outcome of PDAC patients (21–23) but most were based on resectable PDAC and assessed their impact on postoperative mortality. The data on patients with advanced or metastatic PDAC undergoing palliative treatment is scarce (9, 24, 25) and have mainly focused on patient’s survival rather than treatment-related toxicity. Choi et al. (24) have reported that sarcopenia is related with poor survival in metastatic PDAC but did not assess muscle quality. Kim et al. (9) and Rollins et al. (25) have evaluated the impact of skeletal muscle index (SMI) and density (SMD) on the prognosis of metastatic PDAC, however the results were conflicting. It would be meaningful to investigate if the pre-treatment muscle mass and/or quality is associated with the occurrence of early chemotherapy-related toxicity, which could guide tailored patient management and help improving patients’ adherence to treatment. The purpose of this study was to explore the role of baseline CT-assessed muscle mass and myosteatosis in predicting the toxicity during first cycle of the first-line chemotherapy in a large population with metastatic PDAC.

## Materials and Methods

### Patient Selection

**Figure 1:**
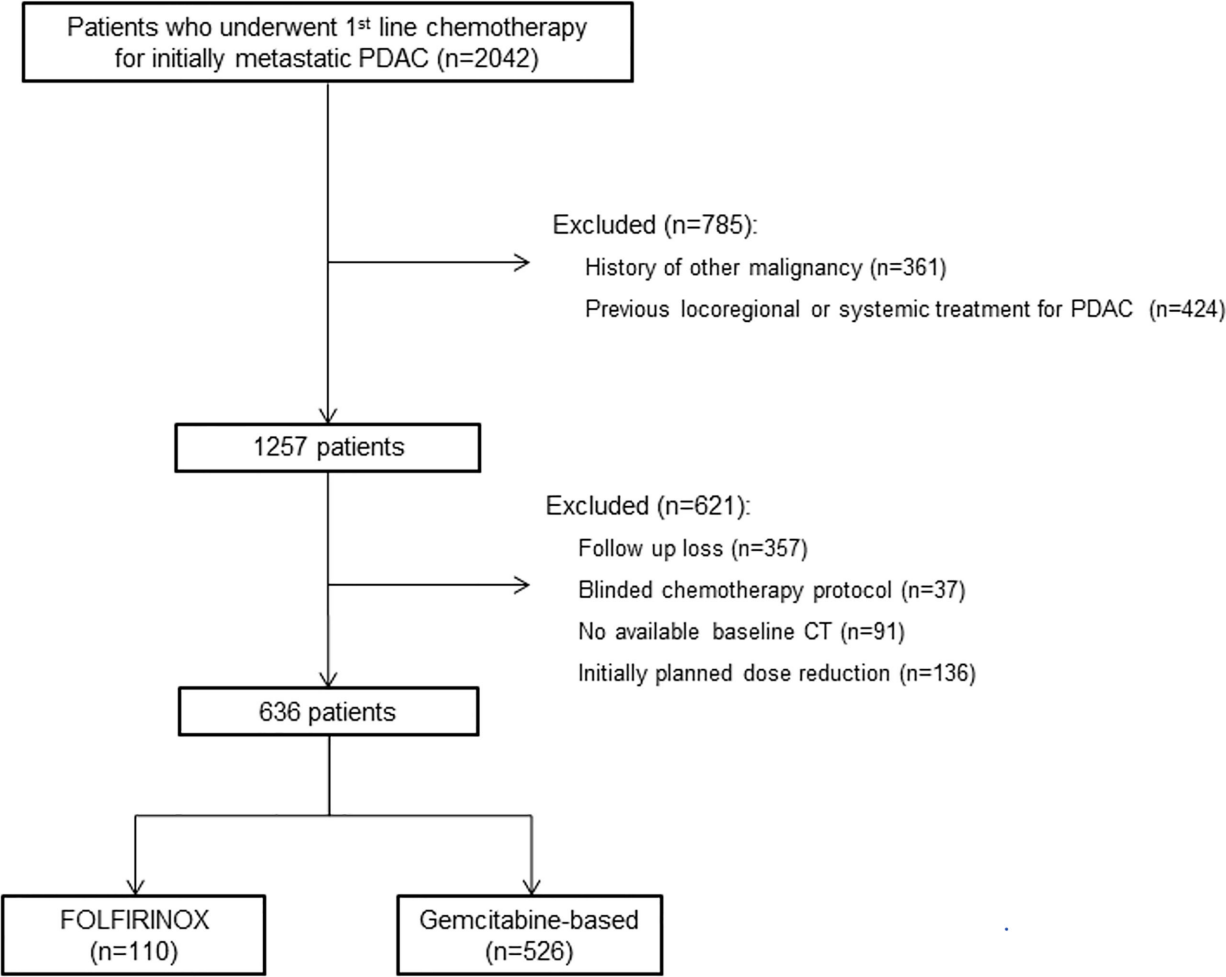
Flow diagram of study population. *PDAC*, pancreatic ductal adenocarcinoma.

This study was approved by the institutional review board of Asan Medical Center, and informed consent was waived due to the retrospective nature of the analysis. Using our institutional database, we identified 2042 consecutive patients who underwent first-line chemotherapy for initially metastatic PDAC from January 2009 to December 2019. Patients who had history of other malignancy or had undergone locoregional or systemic treatment for PDAC were excluded. Among the 1257 patients screened, patients who became lost to follow up before or during the first cycle of first-line chemotherapy, received blinded chemotherapy protocol as parts of the randomized clinical trials, had no available baseline CT, and did not receive standard dose of chemotherapy were excluded. A total of 636 patients were included (**Figure 1**). Baseline clinical data including age, sex, height, weight, body mass index (BMI), body surface area, Eastern Cooperative Oncology Group (ECOG) performance status, and laboratory data including carcinoembryonic antigen (CEA) and carbohydrate antigen 19-9 (CA19-9) were collected from electronic medical records.

### First-Line Chemotherapy and Toxicity Assessment

Patients received either FOLFIRINOX (n=110) or gemcitabine-based treatment (n=526) as a first-line chemotherapy. In FOLFIRINOX, oxaliplatin (85 mg/m^2^), leukovorin (400 mg/m^2^), and irinotecan (180 mg/m^2^) were delivered intravenously on day 1 followed by 400 mg/m^2^ (bolus) and 2400 mg/m^2^ (continuously over a 46-h period) of 5-FU every 2 weeks. Dose modification was made in 56 patients (50.9%) to slightly reduce the dose of irinotecan (120 mg/m^2^ or 150 mg/m^2^) at the treating oncologist’s decision. Among the gemcitabine-based regimens, gemcitabine plus nab-paclitaxel was most common (53.8%; 283/526), followed by gemcitabine alone (30.4%; 160/526), gemcitabine plus erlotinib (9.9%; 52/526) and gemcitabine plus capecitabine (5.9%; 31/526). In all gemcitabine-based regimens, patients received a slow intravenous infusion of gemcitabine (1000 mg/m^2^) on days 1, 8, and 15 with or without combined drugs of a 28-day cycle (every 4 weeks). All chemotherapy-related toxicities were evaluated based on the National Cancer Institute Common Toxicity Criteria for Adverse Events (CTCAE) version 5.0 (26). Toxicity assessments were performed at each scheduled chemotherapy session or whenever clinically indicated by the treating oncologists.

### Endpoint

The primary endpoints were grade 3–4 toxicity based on CTCAE version 5.0 (26) and any treatment-modifying toxicity which led to a dose reduction, delayed administration, drug skip or discontinuation. In both endpoints, toxicities developed only during the first cycle were recorded.

### Body Composition Analysis

Baseline contrast-enhanced abdomen CT images were used to assess body composition. A single, portal venous phase axial CT image at the level of lower endplate of the 3^rd^ lumbar vertebra was used. Details of CT scanning techniques are in **Supplementary Material** and **Supplementary Table 1**. As previously reported, this level is considered the best reference site to assess lean muscle (27) and have shown a better correlation with whole body muscle mass (12) than other methods, including bioelectrical impedance analysis and dual x-ray absorptiometry (28). The cross-sectional areas of total abdominal wall muscle (skeletal muscle area [SMA]; including psoas, paraspinal, transversus abdominis, rectus abdominis, quadratus lumborum, and internal and external obliques), subcutaneous adipose tissue, and visceral adipose tissue were measured with the pre-established thresholds (from -29 to +150 HU for SMA and from -190 to -30 HU for SAT and VAT) (29). The low attenuated muscle area (LAMA) was obtained with the pre-established threshold (from -29 to +29 HU) (18, 30, 31). All segmentation processes were automatically performed using the web-based toolkit program (32), and reviewed and corrected as necessary by a board-certified abdominal radiologist (H.J.P. with 8 years of clinical experience) who were blinded to the patient information.

The body morphometry parameters were normalized by being divided to the patient height squared (cm^2^/m^2^) and reported as indexes including skeletal muscle index (SMI), subcutaneous adipose tissue index (SATI), and visceral adipose tissue index (VATI). LAMA/SMA index was expressed as percentage, i.e., 100 × (LAMA [cm^2^]/SMA [cm^2^]). To identify the presence of low muscle mass, the sex-specific cutoff values were set at SMI less than 41 cm^2^/m^2^ for women, and less than 43 cm^2^/m^2^ (if BMI is less than 25 cm/kg^2^) or less than 53 cm^2^/m^2^ (if BMI is equal or higher than 25 cm/kg^2^) for men (16). Patients with LAMA/SMA index ≥ 20% were regarded as having myosteatosis. To evaluate whether the low muscle mass accompanied by myosteatosis has any impact on toxicity, we also evaluated the presence or absence of low muscle mass with myosteatosis.

### Statistical Analysis

Variables not normally distributed were log-transformed. To identify factors predicting the occurrence of grade 3–4 toxicity or treatment-modifying toxicity, univariate and multivariable binary logistic regression analyses were performed including the candidate predictors selected from all clinical and body composition parameters using bootstrapping with 500 interactions (i.e., selected variables in >50% of the bootstrap models) (33), as recommended by Transparent reporting of a multivariable prediction model for individual prognosis or diagnosis (TRIPOD) statement (34). Missing data were processed with multiple imputation (35) and with the majority method for variable selection (36). To evaluate whether the chemotherapy protocol modifies the association of body morphometry parameters with the occurrence of toxicity, interaction terms (protocol × body morphometry parameters) were included in the regression analysis. The incremental differences between the models in predicting treatment-related toxicities were evaluated using net reclassification improvement (NRI) and integrated discrimination improvement (IDI). The NRI is used to evaluate the net proportion of patients who have been reclassified correctly using the new model (i.e., with body morphometry analysis) relative to the baseline model (i.e., without body morphometry analysis) whereas the IDI measures the improvement in sensitivity of the new model relative to the baseline model, without a loss in specificity (37). All statistical analyses were performed using R version 3.6.1 (R Foundation for Statistical Computing, Vienna, Austria). Two-sided *P* values < 0.05 were considered significant.

## Results

### Patient Characteristics

The patients’ characteristics are summarized in **Table 1**. The median age of the 636 included patients was 60 years, and 58.6% (373/636) were men. Median BMI was 22.6 kg/m^2^. Most of the patients had ECOG performance score of 0–1 (86.6%; 551/636). Low muscle mass was present in 34.0% of patients (216/636). Patients who received FOLFIRINOX were younger than those who received gemcitabine-based chemotherapy (median age, 56 vs. 61; *P* < 0.001). The proportion of men was higher in patients who received FOLFIRINOX (56.7% vs. 68.2%; *P* = 0.03). Patients received gemcitabine-based regimen had a higher CEA and lower hemoglobin level than those who received FOLFIRINOX (CEA median value, 5.0 ng/mL vs. 3.2 ng/mL; *P* = 0.04 and hemoglobin median value, 12.7 g/dl vs. 13.1 g/dl; *P* = 0.01). There was no significant difference in BMI, ECOG performance score, and the prevalence of low muscle mass and no other laboratory data were different between two regimen groups. SMI was higher in patients who received FOLFIRINOX than gemcitabine-based regimen (47.6 cm^2^/m^2^ vs. 45.3 cm^2^/m^2^; *P* = 0.01) but no other body morphometry parameters were different between two groups. The median time from CT scan to the initiation of chemotherapy was 13 days (interquartile range, 9 to 21 days).

**Table 1:** Characteristics of the included patients.

Characteristics	Total(n=636)	Gemcitabine-based (n=526)	FOLFIRINOX(n=110)	*P* value^†^
**Demographics**				
Sex				0.03
Men	373 (58.6%)	298 (56.7%)	75 (68.2%)	
Women	263 (41.4%)	228 (43.3%)	35 (31.8%)	
Age (years)*	60.0 (53.5–67.0)	61.0 (54.0–67.0)	56.0 (49.0–61.0)	<0.001
BMI (kg/m^2^)*	22.6 (20.7–24.3)	22.5 (20.7–24.3)	22.9 (20.8–24.4)	0.40
ECOG				0.57
0	224 (39.3%)	185 (40.2%)	39 (35.5%)	
1	327 (57.4%)	259 (56.3%)	68 (61.8%)	
2	19 (3.3%)	16 (3.5%)	3 (2.7%)	
Laboratory data*				
CEA (ng/mL)	4.6 (2.0–15.1)	5.0 (2.1–16.2)	3.2 (1.7–10.8)	0.04
CA 19-9 (IU/mL)	588.0 (108.0–3211.0)	649.8 (113.7–3395.0)	362.2 (63.7–2477.0)	0.12
WBC (x10^3^/uL)	6.7 (5.4–8.2)	6.7 (5.4–8.2)	6.9 (5.4–8.5)	0.43
Hemoglobin (g/dl)	12.7 (11.7–13.6)	12.7 (11.7–13.6)	13.1 (12.1–14.1)	0.01
Platelet count (x10^3^/uL)	219.0 (175.0–276.5)	221.0 (175.0–279.0)	215.0 (168.0–266.0)	0.38
AST (IU/L)	21.0 (17.0–29.5)	21.0 (16.0–29.0)	21.5 (17.0–35.0)	0.20
ALT (IU/L)	19.0 (12.0–34.0)	19.0 (12.0–34.0)	20.0 (14.0–37.0)	0.17
Total bilirubin (mg/dL)	0.6 (0.4–0.8)	0.6 (0.4–0.8)	0.6 (0.4–0.9)	0.76
ALP (IU/L)	88.5 (65.0–137.5)	89.0 (65.0–138.0)	88.0 (64.0–137.0)	0.65
**Body morphometry parameters**				
SATI (cm^2^/m^2^)*	39.7 (26.2–55.7)	39.8 (26.6–56.3)	38.9 (24.8–52.8)	0.68
VATI (cm^2^/m^2^)*	31.4 (19.0–45.9)	30.9 (19.0–45.8)	32.2 (19.3–7.2)	0.48
SMI (cm^2^/m^2^)*	45.8 (40.5–51.6)	45.3 (40.2–51.2)	47.6 (42.4–53.0)	0.01
LAMI (cm^2^/m^2^)*	9.7 (7.4–12.9)	9.8 (7.5–13.1)	9.1 (7.0–12.5)	0.17
LAMI/SMI ≥ 20%	353 (55.5%)	298 (56.7%)	55 (50.0%)	0.20
Low muscle mass^‡^	216 (34.0%)	186 (35.4%)	30 (27.3%)	0.13
Low muscle mass with myosteatosis^§^	152 (23.9%)	130 (24.7%)	22 (20.0%)	0.29

*Median values with interquartile range or frequency in parentheses. Otherwise, data are number with percentage in parentheses.

^†^Comparison between gemcitabine-based group and FOLFIRINOX group.

^‡^SMI less than 41 cm^2^/m^2^ for women, and less than 43 cm^2^/m^2^ (if BMI is less than 25 cm/kg^2^) or less than 53 cm^2^/m^2^ (if BMI is equal or higher than 25 cm/kg^2^) for men.

^§^Presence of both low muscle mass and LAMI/SMI ≥ 20%.

ALP, alkaline phosphatase; ALT, alanine aminotransferase; AST, aspartate aminotransferase; BMI, Body mass index; CA 19-9, carbohydrate antigen 19-9; CEA, carcinoembryonic antigen; ECOG, Eastern Cooperative Oncology Group performance status score; LAMI, low attenuated muscle index; SATI, subcutaneous adipose tissue index; SMI, skeletal muscle index; VATI, visceral adipose tissue index; WBC, white blood cell.

### Toxicity During Chemotherapy

Among the 636 included patients, 160 patients (25.2%) experienced at least one grade 3–4 toxicity during the first cycle of the first-line chemotherapy. Neutropenia was the most common type of grade 3–4 toxicity (75.0%; 120/160), followed by nausea (14.4%, 23/160) and thrombocytopenia (5.6%, 9/160). Treatment-modifying toxicity occurred in 247 patients (38.8%). In patients who experienced treatment-modifying toxicity, treatment modification included dose reduction in 100 patients (15.7%), delayed administration in 63 patients (9.9%), both dose reduction and delayed administration in 34 patients (5.3%), drug skip in 35 patients (5.5%) and discontinuation of chemotherapy in 15 patients (2.4%). Details of the toxicity occurred in patients are provided in **Table 2**. Toxicity according to each gemcitabine-based regimen is shown in **Supplementary Table 2**.

**Table 2:** Toxicity during the first cycle of first-line chemotherapy.

Toxicities	Gemcitabine-based (n=526)		FOLFIRINOX (n=110)	
	Grade 3 or 4 toxicity	Treatment-modifying toxicity*	Grade 3 or 4 toxicity	Treatment-modifying toxicity^†^
No. of patients	118 (22.4%)	204 (38.8%)	42 (38.2%)	43 (39.1%)
Neutropenia	93 (78.8%)	108 (52.9%)	28 (66.7%)	30 (69.8%)
Nausea	9 (7.6%)	19 (9.3%)	14 (33.3%)	6 (14.0%)
Thrombocytopenia	9 (7.6%)	26 (12.7%)	0	0
Rash maculo-papular	4 (3.4%)	8 (3.9%)	0	0
ALT or AST increased	3 (2.5%)	4 (2.0%)	1 (2.4%)	1 (2.3%)
Anemia	1 (0.8%)	6 (2.9%)	0	0
Diarrhea	1 (0.8%)	1 (0.5%)	0	0
Fatigue	1 (0.8%)	12 (5.9%)	1 (2.4%)	1 (2.3%)
Fever	0	4 (2.0%)	0	0
Acute kidney injury	0	1 (0.5%)	0	0
Other toxicity^‡^	0	1 (0.5%)	0	3 (7.0%)

The sum of percentages may exceed 100% as some patients had two or more types of toxicity.

*Dose reduction in 89 patients (43.6%), delayed administration in 40 patients (19.6%), both dose reduction and delayed administration in 30 patients (14.7%), drug skip in 34 patients (16.7%) and discontinuation of chemotherapy in 11 patients (5.4%).

^†^Dose reduction in 11 patients (25.6%), delayed administration in 23 patients (53.5%), both dose reduction and delayed administration in 4 patients (9.3%), drug skip in 1 patient (2.3%) and discontinuation of chemotherapy in 4 patients (9.3%).

^‡^Other chemotherapy induced toxicity included non-cardiac chest pain, gastritis, encephalopathy, and hearing impaired.

ALT, alanine aminotransferase; AST, aspartate aminotransferase.

### Factors Influencing the Occurrence of Toxicity

**Figure 2:**
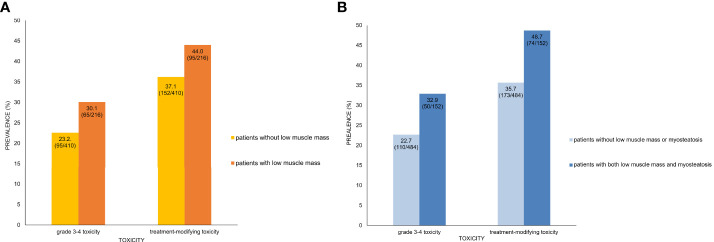
Prevalence of toxicity and presence of low muscle mass only **(A)** and both low muscle mass and myosteatosis **(B)**. **(A)** In patients with low muscle mass, 30.1% and 44.0% showed grade 3–4 toxicity and treatment-modifying toxicity, respectively. In contrast, patients without low muscle mass had lower prevalence of severe toxicity (23.2%) and DLT (37.1%). **(B)** Patients who had both low muscle mass and myosteatosis had higher prevalence of grade 3–4 toxicity (32.9% vs. 22.7%) and treatment-modifying (48.7% vs. 35.7%) than those without low muscle mass or myostatosis. Note that the inter-group difference of toxicity prevalence is larger between patients with and without both low muscle mass and myosteatosis than between those with and without low muscle mass alone.

Results of the univariate and multivariable analyses on the occurrence of grade 3–4 toxicity is shown in **Table 3**. In univariate analysis, the type of chemotherapy regimen, white blood cell (WBC) count, platelet count, LAMI, presence of low muscle mass, and presence of both low muscle mass and myosteatosis had association with the occurrence of grade 3–4 toxicity. Multivariable analysis including the factors selected from 500 bootstrap samples (chemotherapy regimen, WBC count, and the presence of both low muscle mass and myosteatosis) revealed that all the input variables influenced the occurrence of grade 3–4 toxicity (odds ratio [OR] 2.46 [95% CI 1.55–3.91] for chemotherapy regimen [reference: gemcitabine-based]; OR 0.74 [95% CI 0.67–0.82] for WBC count; and OR 1.73 [95% CI 1.14–2.63] for low muscle mass with myosteatosis). While grade 3–4 toxicity occurred in 65 among 216 patients with low muscle mass (12.6%), 50 out of 152 patients with both low muscle mass and myosteatosis experienced grade 3–4 toxicity (32.9%) (**Figure 2**). When testing the interaction between chemotherapy regimen and the presence of low muscle mass with myosteatosis, there was no association between those two parameters (*P* value for interaction = 0.85). By adding the presence of low muscle mass and myosteatosis to the WBC, the model achieved a net improvement in its classification and discrimination performance compared with the use of WBC alone (NRI, 0.196 [95% CI, 0.035–0.358, *P* = 0.02]; IDI, 0.012 [95% CI, 0.003–0.021, *P* = 0.01]). This suggested that there is a significant incremental value in using body composition analysis to predict grade 3–4 toxicity.

**Table 3:** Univariate and multivariable analyses of the occurrence of grade 3–4 toxicity.

Variables	Univariate analysis		Multivariable analysis	
	Odds ratio (95% CI)	*P* value	Odds ratio (95% CI)	*P* value
Sex				
Men	Reference			
Women	1.07 (0.74–1.53)	0.73		
Age (years)	0.99 (0.97–1.01)	0.39		
Chemotherapy regimen				
Gemcitabine-based	Reference		Reference	
FOLFIRINOX	2.14 (1.38–3.30)	0.001	2.46 (1.55–3.91)	<0.001
BMI (kg/m^2^)	0.96 (0.90–1.02)	0.16		
ECOG		0.20		
0	Reference			
1	0.98 (0.67–1.43)	0.91		
2	0.31 (0.07–1.37)	0.12		
Log CEA	0.94 (0.84–1.04)	0.22		
Log CA19-9	0.99 (0.93–1.05)	0.73		
WBC (x10^3^/uL)	0.75 (0.68–0.83)	<0.001	0.74 (0.67–0.82)	<0.001
Hemoglobin (g/dl)	1.09 (0.96–1.23)	0.18		
Platelet count (x10^3^/uL)	0.994 (0.992–0.997)	<0.001		
AST (IU/L)	0.99 (0.98–1.01)	0.30		
ALT (IU/L)	0.99 (0.99–1.00)	0.09		
Total bilirubin (mg/dL)	1.14 (0.79–1.64)	0.48		
ALP (IU/L)	0.998 (0.996–1.00)	0.13		
SATI (cm^2^/m^2^)	1.00 (0.99–1.01)	0.55		
VATI (cm^2^/m^2^)	0.99 (0.98–1.00)	0.06		
SMI (cm^2^/m^2^)	0.99 (0.97–1.01)	0.40		
LAMI (cm^2^/m^2^)	0.94 (0.90–0.98)	0.01		
LAMI/SMI		0.61		
< 20%	Reference			
≥ 20%	0.91 (0.64–1.30)			
Low muscle mass*	1.47 (1.02–2.13)	0.04		
Low muscle mass with myosteatosis^†^	1.67 (1.12–2.49)	0.01	1.73 (1.14–2.63)	0.01

*SMI less than 41 cm^2^/m^2^ for women, and less than 43 cm^2^/m^2^ (if BMI is less than 25 cm/kg^2^) or less than 53 cm^2^/m^2^ (if BMI is equal or higher than 25 cm/kg^2^) for men.

^†^Presence of both low muscle mass and LAMI≥20% of SMI.

ALP, alkaline phosphatase; ALT, alanine aminotransferase; AST, aspartate aminotransferase; BMI, Body mass index; CA 19-9, carbohydrate antigen 19-9; CEA, carcinoembryonic antigen; CI, confidence interval; ECOG, Eastern Cooperative Oncology Group performance status score; LAMI, low attenuated muscle index; SATI, subcutaneous adipose tissue index; SMI, skeletal muscle index; VATI, visceral adipose tissue index; WBC, white blood cell.

**Figure 3:**
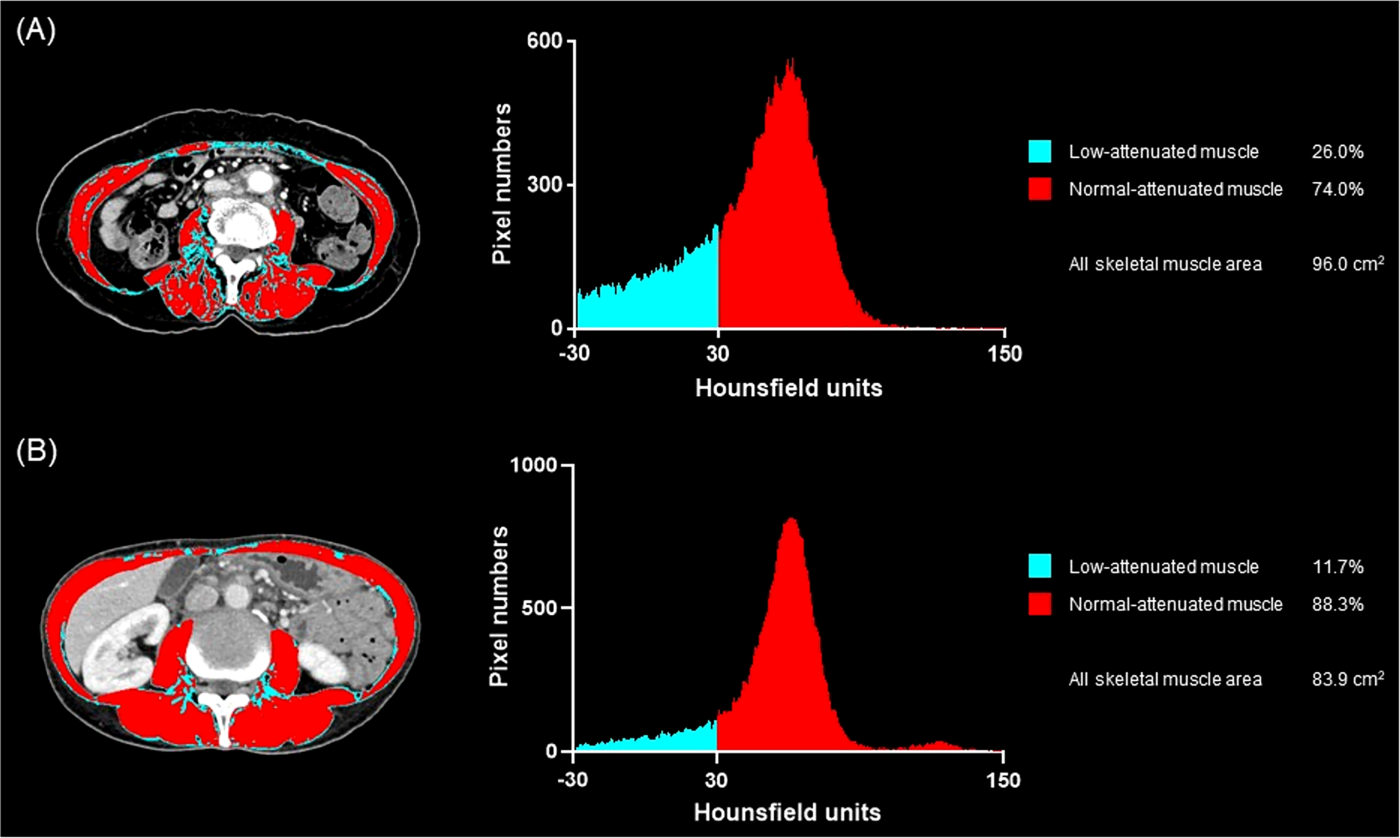
Representative cases of low muscle mass with or without myosteatosis and the occurrence of treatment-related toxicity. **(A)** A 66-year-old woman treated with gemcitabine monotherapy. She had pre-treatment BMI of 21.2 kg/m^2^ and the baseline skeletal muscle index of 40.0, suggesting that her muscle mass was low. The muscle quality map and histogram analysis show the low-attenuated muscle area (blue) is 26.0% of the skeletal muscle area (blue plus red) suggesting that she also had myosteatosis. She experienced grade 3 neutropenia at day 15 after treatment initiation, and the chemotherapy schedule was delayed. **(B)** A 55-year-old woman who underwent gemcitabine plus nab-paclitaxel, in whom the baseline BMI was 15.0 kg/m^2^ and the skeletal muscle index was 33.3, suggesting low muscle mass, more severe than the patient in **(A)**. The muscle quality map shows the low-attenuated muscle area (blue) is only 11.7% of the skeletal muscle area (blue plus red) suggesting the absence of myosteatosis. She did not experience any toxicity during the first cycle of chemotherapy.

The factors regarding the occurrence of treatment-modifying toxicity are shown in **Table 4**. In univariate analysis, WBC count, platelet count, alanine aminotransferase, and the presence of both low muscle mass and myosteatosis were associated with the occurrence of treatment-modifying toxicity. WBC count and the presence of low muscle mass with myosteatosis were the factors included in multivariable analysis selected from 500 bootstrap samples. The two factors had significant influence on the occurrence of treatment-modifying toxicity: OR of 0.996 (95% CI 0.99–0.998) for platelet and OR of 1.83 (95% CI 1.26–2.66) for the presence of both low muscle mass and myosteatosis. In terms of predicting treatment-modifying toxicities, inclusion of the presence of a low muscle mass and myosteatosis to the platelet count improved the model’s classification and discrimination ability compared with the use of the platelet count alone (NRI, 0.198 [95% CI, 0.059–0.337, *P* = 0.005]; IDI, 0.016 [95% CI, 0.006–0.026], *P* = 0.002). Treatment-modifying toxicity occurred in 95 patients among 216 patients with low muscle mass (44.0%). Of 152 patients with both low muscle mass and myosteatosis, 74 patients experienced treatment-modifying toxicity (48.7%) (**Figure 2**). The representative cases are shown in **Figure 3**.

**Table 4:** Univariate and multivariable analyses of the occurrence of treatment-modifying toxicity.

Variables	Univariate analysis		Multivariable analysis	
	Odds ratio (95% CI)	*P* value	Odds ratio (95% CI)	*P* value
Sex				
Men	Reference			
Women	1.17 (0.85–1.62)	0.33		
Age (years)	1.01 (1.00–1.03)	0.16		
Chemotherapy regimen				
Gemcitabine-based	Reference			
FOLFIRINOX	1.01 (0.67–1.54)	0.95		
BMI (kg/m^2^)	0.98 (0.93–1.03)	0.47		
ECOG				
0	Reference	0.99		
1	1.00 (0.70–1.41)	0.98		
2	1.08 (0.42–2.80)	0.87		
Log CEA	0.94 (0.86–1.03)	0.18		
Log CA19-9	0.98 (0.93–1.04)	0.51		
WBC (x10^3^/uL)	0.88 (0.82–0.95)	0.001		
Hemoglobin (g/dl)	1.00 (0.90–1.11)	0.99		
Platelet count (x10^3^/uL)	0.996 (0.994–0.998)	<0.001	0.996 (0.994–0.998)	<0.001
AST (IU/L)	0.99 (0.98–1.00)	0.12		
ALT (IU/L)	0.99 (0.99–1.00)	0.03		
Total bilirubin (mg/dL)	1.03 (0.74–1.44)	0.86		
ALP (IU/L)	0.999 (0.997–1.00)	0.20		
SATI (cm^2^/m^2^)	1.00 (0.99–1.01)	0.94		
VATI (cm^2^/m^2^)	1.00 (0.99–1.01)	0.98		
SMI (cm^2^/m^2^)	0.99 (0.97–1.01)	0.29		
LAMI (cm^2^/m^2^)	0.99 (0.95–1.03)	0.56		
LAMI/SMI		0.26		
< 20%	Reference			
≥ 20%	1.20 (0.87–1.66)			
Low muscle mass*	1.38 (0.99–1.93)	0.06		
Low muscle mass with myosteatosis^†^	1.71 (1.18–2.47)	0.01	1.83 (1.26–2.66)	0.002

*SMI less than 41 cm^2^/m^2^ for women, and less than 43 cm^2^/m^2^ (if BMI is less than 25 cm/kg^2^) or less than 53 cm^2^/m^2^ (if BMI is equal or higher than 25 cm/kg^2^) for men.

^†^Presence of both low muscle mass and LAMI≥20% of SMI.

ALP, alkaline phosphatase; ALT, alanine aminotransferase; AST, aspartate aminotransferase; BMI, Body mass index; CA 19-9, carbohydrate antigen 19-9; CEA, carcinoembryonic antigen; CI, confidence interval; ECOG, Eastern Cooperative Oncology Group performance status score; LAMI, low attenuated muscle index; SATI, subcutaneous adipose tissue index; SMI, skeletal muscle index; VATI, visceral adipose tissue index; WBC, white blood cell.

## Discussion

In this study, we evaluated the influence of low muscle mass and myosteatosis on the occurrence of toxicity during the first cycle of first-line chemotherapy in 636 patients with initially metastatic PDAC. Among the various body composition parameters included in the analysis, the presence of both low muscle mass and myosteatosis was an independent risk factor for the development of early chemotherapy-related toxicity, both in terms of grade 3–4 toxicity and treatment-modifying toxicity. These suggest that comprehensive assessment of both muscle mass and quality would be important to predict early treatment-related adverse events, which would help stratifying patients at risk for developing toxicity and enable tailored approach to minimize treatment interruption.

In our study, although low muscle mass had a significant association with grade 3–4 toxicity in univariate analysis, it was not selected as a significant predictor on feature selection and multivariable analysis. Although low muscle mass has been suggested as a predictor of survival in PDAC patients (21, 23, 24), it may be insufficient to predict early toxicity of chemotherapy with the presence of low muscle mass alone. This result is consistent with Rollins et al.’s study (25) reporting that sarcopenia alone did not have significant difference in overall survival in unresectable PDAC patients. Rather, the presence of both low muscle mass and myosteatosis appeared to be a significant factor predicting both grade 3–4 toxicity and treatment-modifying toxicity in our study, suggesting that decreased muscle mass accompanied by impaired muscle quality caused by intramuscular fat infiltration may be a predictor of early chemotherapy-related toxicity. This is in line with the prior study of PDAC with smaller sample size (9) which reported that metastatic PDAC patients with sarcopenia and low SMD experienced severe chemotherapy toxicity., also with studies of other cancer types (38, 39). Another recent study on elderly cancer patients showed that SMD was more associated with physical function than skeletal muscle mass (40). More fatty infiltration of muscle represents lower level of physical activity (41) and is associated with more inflammation (42), which possibly increases the likelihood of developing higher incidence of treatment-related toxicity in sarcopenic patients.

CTCAE is the most widely used criteria to assess treatment-induced toxicities in cancer patients (26), and grade 3–4 toxicity defined by CTCAE is commonly used to report the safety profile of treatment (43). However, in clinical practice, the occurrence of multiple lower grade toxicities is often considered clinically relevant as they also cause deterioration in patients’ quality of life, decrease adherence, and lead to changes in clinical decision-making. Treatment-modifying toxicity has been proposed for an additional or even alternative concept in evaluating adverse effects, and daily low-grade toxicities are of increasing clinical significance in current cancer trials (44, 45). In our study, we used both grade 3–4 toxicity and treatment-modifying toxicity as study endpoints for comprehensive assessment of treatment-related adverse events. The presence of both low muscle mass and myosteatosis had significant influence on toxicity occurrence, consistently in both grade 3–4 toxicity and treatment-modifying toxicity (OR 1.73, 95% CI 1.14–2.63 for grade 3–4 toxicity and OR 1.83 with 95% CI 1.26-2.66 for treatment-modifying toxicity), suggesting that it may be a robust predictor of toxicity beyond the current debate on the definition of toxicity.

Regimen type (gemcitabine-based vs. FOLFIRINOX) also showed significant association with grade 3–4 toxicity on multivariable analysis. Patients receiving FOLFIRINOX had a significantly higher incidence of grade 3–4 toxicity (38.2%) than gemcitabine-based regimens (22.4%). This is in line with previous studies reporting the higher toxicity of FOLFIRINOX than gemcitabine-based treatment (46, 47). However, regimen type was not a significant predictor of treatment-modifying toxicity. We infer that assessment of treatment-modifying toxicity would have been influenced by different practice patterns of treating oncologists. We further investigated if the regimen type influenced on the impact of low muscle mass with myosteatosis on the occurrence of either grade 3–4 toxicity or treatment-modifying toxicity by including the interaction term in the multivariable analyses, and there was no association between parameters, suggesting that the impact of low muscle mass with myosteatosis on the toxicity is robust, regardless of the regimen type.

There are several limitations in our study. First, this is a retrospective single-center study, having potential risk of biases, although efforts were made to ensure the data were as complete and accurate as possible. Second, to define myosteatosis, we used an arbitrary criterion (i.e., LAMA/SMA ≥ 20%), which might limit the generalizability of our result. Prior studies have used SMD (the average radiodensity of the whole muscle areas) to assess myosteatosis (9, 25), but SMD has several pitfalls; it has not been well validated, is easily influenced by various CT scanner, techniques, and image artifacts, and does not provide detailed quantity of muscles with and without myosteatosis. We tried to stratify and assess the distribution of muscle components according to the degree of fat infiltration, which would facilitate more precise evaluation of the myosteatosis and muscle quality. Third, we excluded patients who received chemotherapy other than gemcitabine-based and FOLFIRINOX. Most of these excluded regimens were rarely used, less than five in total number of patients. Excluding those were to maintain study homogeneity yet focusing the most widely used two standard regimens in metastatic PDAC. Despite these limitations, this is the largest study to date on the impact of baseline body composition in patients with metastatic PDAC.

In conclusion, our result suggests the presence of both low muscle mass and myosteatosis on baseline CT may be used to predict treatment-related toxicity during the first cycle of first-line chemotherapy in patients with metastatic PDAC. Careful selection of regimen and dose with further medical support for those having both low muscle mass and myosteatosis at baseline would be helpful to minimize treatment interruption and to improve patients’ compliance.

## Data Availability Statement

The original contributions presented in the study are included in the article/**Supplementary Material**. Further inquiries can be directed to the corresponding author.

## Ethics Statement

The studies involving human participants were reviewed and approved by Institutional Review Board of Asan Medical Center. Written informed consent for participation was not required for this study in accordance with the national legislation and the institutional requirements.

## Author Contributions

HP contributed to the study concept and design. SH, KK, and HP acquired and analyzed the data. YK, SK, HJ, and JL performed and analyzed automatized program to obtain body parameters from CT images. SP performed the statistical analysis. SH, KK, and HP drafted the manuscript. CY made critical revisions to the manuscript. All authors contributed to the article and approved the submitted version.

## Funding

This study was supported by a grant of the Korea Health Technology R&D Project through the Korea Health Industry Development Institute (KHIDI), funded by the Ministry of Health & Welfare, Republic of Korea (grant number: HI18C1216), and a grant of the National Research Foundation of Korea(NRF) funded by Korea government(MSIT) (No. 2021R1C1C1010138).

## Conflict of Interest

The authors declare that the research was conducted in the absence of any commercial or financial relationships that could be construed as a potential conflict of interest.

## Publisher’s Note

All claims expressed in this article are solely those of the authors and do not necessarily represent those of their affiliated organizations, or those of the publisher, the editors and the reviewers. Any product that may be evaluated in this article, or claim that may be made by its manufacturer, is not guaranteed or endorsed by the publisher.
