# Supplementary material for: Impact of Baseline Muscle Mass and Myosteatosis on the Development of Early Toxicity During First-Line Chemotherapy in Patients With Initially Metastatic Pancreatic Cancer
Source: Front Oncol. 2022 May 20;12:878472. doi: 10.3389/fonc.2022.878472 (PMC9163383; doi:10.3389/fonc.2022.878472)
Supplement: Supplementary file 1 [file DataSheet_1.docx]

***Supplementary Material***

**CT acquisition**

Contrast-enhanced abdomen CT including portal venous phase (PVP) were obtained for all patients. CT scanners from various manufacturers (LightSpeed 16, LightSpeed plus, and LightSpeed VCT scanners [GE Healthcare, Milwaukee, WI, USA], Sensation 16, Somatom Definition, Somatom Definition flash, and Somatom Definition AS + scanners [Siemens Medical Systems, Erlangen, Germany], Aquilion scanner [Toshiba, Tokyo, Japan], Ingenuity, iCT 256 scanners [Philips Healthcare, Amsterdam, Netherlands], ECLOS [Hitachi Medical Corporation, Tokyo, Japan], Aquilion Start [Canon Medical Systems, Otawara, Japan] and SCT-7800TE [Shimadzu Corporation, Tokyo, Japan] were used. Most CT images were obtained using a 16-detector or higher CT system (n=466 [73.3%]). Most of the CT images were acquired using 120 kVp (n=338 [53.1%]), followed by 130-140 kVp (n=222 [34.9%]) and 80-110 kVp (n=76 [11.9%]). PVP imaging was performed 70–80 second after intravenous injection of the contrast agent. The images were reconstructed in the axial plane and ranged from 2.5 to 5 mm in thickness at 2.5 to 5 mm intervals. Most of the CT images had slice thickness of 2-4 mm (n=331 [52.0%]), followed by 5 mm (n=143 [22.5%]) and 6-7 mm (n=2 [0.3%]). 160 CT images had no information of detector number and slice thickness. Images were reconstructed using filtered back projection (B30f, B30s, B41f, B41s) or iterative reconstruction (I30s, I30f). Pixel size ranged from 0.53 mm to 0.98 mm.

**Table S1. CT imaging techniques**

| **CT techniques** | **Patient number (n=636)** | |
| --- | --- | --- |
| CT detector configuration, n (%) |  |  |
| < 16 detectors | 10 | (1.6) |
| 16–64 detectors | 110 | (17.3) |
| ≥ 64 detectors | 356 | (56.0) |
| Unknown | 160 | (25.2) |
| Tube voltage, n (%) |  |  |
| 80–110 kVp | 76 | (11.9) |
| 120 kVp | 338 | (53.1) |
| 130–140 kVp | 222 | (34.9) |
| Slice thickness, n (%) |  |  |
| 2–4 mm | 331 | (52.0) |
| 5 mm | 143 | (22.5) |
| 6–7 mm | 2 | (0.3) |
| Unknown | 160 | (25.2) |
| CT vendors, n (%) |  |  |
| GE | 332 | (52.2) |
| Siemens | 259 | (40.7) |
| Toshiba | 21 | (3.3) |
| Philips | 20 | (3.2) |
| Others^*^ | 4 | (0.6) |
| Beam pitch^†^ | 0.6–1.4 | |

Unless otherwise indicated, data are numbers, with percentages in parentheses

*Canon, Shimadzu and Hitachi Medical Corporation.

^†^Data are ranges.

**Table S2. Treatment-related toxicity during the first cycle according to the gemcitabine-based regimen**

| **Toxicity** | **GEMABRA (n=283)** | **GEM (n=160)** | **GT (n=52)** | **XELGEM (n=31)** |
| --- | --- | --- | --- | --- |
| Neutropenia | 52 (18.4%) | 34 (21.3%) | 13 (25.0%) | 10 (32.3%) |
| Nausea | 14 (4.9%) | 5 (3.1%) | 1 (1.9%) | 0 |
| Thrombocytopenia | 13 (4.6%) | 8 (5.0%) | 2 (3.8%) | 4 (12.9%) |
| Rash maculo-papular | 3 (1.1%) | 2 (1.3%) | 2 (3.8%) | 1 (3.2%) |
| ALT or AST increased | 3 (1.1%) | 1 (0.6%) | 0 | 0 |
| Anemia | 4 (1.4%) | 1 (0.6%) | 1 (1.9%) | 0 |
| Diarrhea | 1 (0.4%) | 0 | 0 | 0 |
| Fatigue | 7 (2.5%) | 2 (1.3%) | 1 (1.9%) | 1 (3.2%) |
| Fever | 1 (0.4%) | 2 (1.3%) | 0 | 1 (3.2%) |
| Acute kidney injury | 1 (0.4%) | 0 | 0 | 0 |
| Other toxicity^*^ | 0 | 0 | 1 (1.9%) | 0 |

Toxicity includes grade 3–4 or treatment-modifying toxicity.

The sum of percentages may exceed 100 as some patients had two or more type of toxicity.

*Other chemotherapy induced toxicity included non-cardiac chest pain.

*ALT,* alanine aminotransferase*; AST;* aspartate aminotransferase*; GEM*, gemcitabine alone; *GEMABRA*, gemcitabine plus nab-paclitaxel; *GT*, gemcitabine plus erlotinib; *XELGEM*, gemcitabine plus capecitabine
